# Prevalence of and Gender Differences in Psychiatric Disorders among Juvenile Detainees in South Korea: A Comparative Study

**DOI:** 10.3390/medicina59122068

**Published:** 2023-11-23

**Authors:** Bum-Sung Choi, Bongseog Kim

**Affiliations:** 1 Department of Psychiatry, Pusan National University Yangsan Hospital, Pusan National University School of Medicine and Medical Research Institute, 20 Geumo-ro, Mulgeum-eup, Yangsan 50612, Republic of Korea; hosei27@naver.com; 2 Department of Psychiatry, Sanggye Paik Hospital, Inje University College of Medicine, 1342 Dong-il Street, Seoul 01757, Republic of Korea

**Keywords:** juvenile detainees, psychiatric disorder, conduct disorder

## Abstract

*Background and Objectives*: High rates of psychiatric disorders and comorbidities have been reported in juvenile detainees, which have been associated with repeat offenses. However, research into this topic has been limited to Asian countries. This study aimed to examine the prevalence of psychiatric disorders and sexual differences among juvenile detainees in a detention center in South Korea. *Materials and Methods*: The participants comprised 54 males and 46 females, with a minimum intelligence score of 80. Psychiatric diagnosis was determined using the Mini-International Neuropsychiatric Interview for Children and Adolescents (MINI-KID). The Massachusetts Youth Screening Instrument-Version 2 (MAYSI-2) was used to investigate gender differences. *Results*: Using the MINI-KID, the most frequent diseases were conduct disorder (CD), alcohol dependence, suicidal tendency, and attention-deficit/hyperactivity disorder (ADHD), with statistically significant differences between men and women. Only alcohol abuse was higher in males, while the rest were higher in females. The items with a statistically significant gender difference in MAYSI-2 were alcohol/drug use, feeling depressed/anxious, somatic complaints, suicidal ideation, and traumatic experiences. All items for which gender difference was statistically significant were higher in the proportion of women. *Conclusions*: Juvenile detainees exhibit high rates of psychiatric disorders and comorbidities. CDs, alcohol dependence, and ADHD are the most common psychiatric disorders among juvenile detainees in South Korea. Assessment of and intervention in psychiatric disorders may help prevent further offenses. These findings highlight the importance of diagnosing and intervening in psychiatric disorders within juvenile detention systems.

## 1. Introduction

The number of youths in the juvenile system with psychiatric disorders presents a major public health problem [1]. Over the past decade, several studies have documented the burden of mental health needs in juvenile justice populations [2,3]. Several studies found high rates of psychiatric disorders in detained adolescent offenders, ranging from 70% to 90%, who suffer from at least one psychiatric disorder [2,4]. This rate is at least three times higher than that of psychiatric disorders in the general adolescent population [5,6]. The young offender population has extensive comorbidities, with 60% having two or more disorders [7,8]. Girls constitute an increasing percentage of the juvenile justice population [9]. Previous studies indicated that many female offenders have comorbid psychiatric disorders that require mental health services. Ariga et al. suggested that delinquent activity among female juvenile offenders is associated with traumatic events and psychiatric symptoms [10]. In contrast to boys entering the legal system, girls are most often nonviolent offenders [11] who present with many psychosocial symptoms, such as abuse, violence, family conflict, pregnancy, and school failure [9,12]. Research into the prevalence of psychiatric disorders among detained adolescents is still limited compared with analogous research into adults. Few studies on psychiatric disorders in juvenile justice settings have published results based on sex and race or ethnicity. Furthermore, limited studies have been published on attention-deficit/hyperactivity disorder (ADHD) in adult inmates, and even fewer studies have considered ADHD in adult inmates by gender. Adolescent girls in the juvenile justice system with mental health problems comprise a particularly vulnerable population that requires early identification and intervention to prevent the development of future problem behaviors [13]. Despite the high rate of psychiatric illnesses among juvenile offenders, research into the psychiatric health of this population and gender differences in Asian countries, including South Korea, is limited. To our knowledge, no South Korean study has estimated the prevalence of psychiatric disorders among juvenile offenders using the MINI-KID. This study aimed to investigate the prevalence of psychiatric disorders among juvenile detainees by sex in South Korea and to assess the patterns of psychopathology. We hypothesized that female juvenile delinquents have higher rates of psychological symptoms than male juvenile delinquents.

## 2. Materials and Methods

### 2.1. Participants and Procedure

Participants were recruited from male and female juvenile detention centers in Seoul, South Korea. According to Article 32, Section 3 of the South Korean Juvenile Act, juvenile offenders are assigned one of ten distinct dispositions following their court trials. This study focused on dispositions numbered 8 to 10, which prescribe various lengths of detention. We excluded detainees sentenced to the 8th disposition, which orders detainment for less than 1 month, and the detainees sentenced to a 6-month (9th disposition) or a 2-year (10th disposition) detainment were included. This study included 54 males and 46 females with an intelligence quotient (IQ) of 80 or higher, with a total of 100 participants. The age range of the participants was 14 to 18 years. Participants were eligible for inclusion in the study regardless of psychiatric diagnosis, degree of drug or alcohol intoxication, or fitness to stand trial. The exclusion criteria included refusal or inability to cooperate or understand the study procedures. Written informed consent was obtained from all participants after the study procedure was explained. The study protocol was approved by the Institutional Review Board of Sanggye Paik Hospital (IRB No. SGPAIK 2015-06-022-002).

### 2.2. Measures

#### 2.2.1. General

The interviewers assessed the demographic characteristics of the participants. The interviewers recorded information related to age, history, and IQ, which were measured in a juvenile classification home. Psychiatric diagnoses were confirmed using the Mini-International Neuropsychiatric Interview for Children and Adolescents (MINI-KID, 6.0). To identify youth with acute mental, emotional, or behavioral problems, the participants were asked to complete the Massachusetts Youth Screening Instrument for Mental Health Needs of Juvenile Justice Youths (MAYSI-2).

#### 2.2.2. The Mini-International Neuropsychiatric Interview for Children and Adolescents

The Mini-International Neuropsychiatric Interview for Children and Adolescents (MINI-KID) is a short, structured clinical diagnostic interview designed to assess the presence of DSM-IV and ICD-10 psychiatric disorders in children and adolescents [14]. The main aim of the MINI-KID is to diagnose existing symptoms of mental disorders. Furthermore, it provides past and lifetime diagnoses clinically relevant to the current diagnosis (e.g., for major depression and manic episodes, as well as a lifetime diagnosis for psychotic disorders and antisocial personality disorder) [14]. Moreover, the MINI-KID allows for the assessment of current and lifetime suicidality [14].

#### 2.2.3. The Massachusetts Youth Screening Inventory

The Massachusetts Youth Screening Instrument for Mental Health Needs of Juvenile Justice Youths (MAYSI-2) is a 52-item self-report true-false measure used in juvenile justice programs to identify youth with acute mental, emotional, or behavioral problems [15]. MAYSI-2 provides reliable and valid numerical indices of several mental health concerns on seven factor analytically derived subscales, namely, alcohol/drug use, angry/irritable, depressed/anxious, somatic complaints, suicidal ideation, thought disturbances, and traumatic experiences [16,17]. Thought disturbance appeared for boys but was not interpretable for girls. Thus, MAYSI-2 has seven subscales for boys and six for girls with good internal consistency, retest reliability, and concurrent validity [16,17]. Although MAYSI-2 was designed to assess acute conditions in a juvenile justice setting, it correlates significantly with several scales from the Child Behavioral Checklist (CBCL) and the Youth Self-Report (YSR) [15,18].

### 2.3. Statistical Analysis

Demographic and clinical characteristics were compared between male and female patients using independent *t*-tests for continuous variables and chi-square or Fisher’s exact tests for categorical variables. All statistical analyses were performed using the SPSS software (ver. 22.0; SPSS Inc., Chicago, IL, USA), and a two-tailed *p*-value < 0.05 was considered significant.

## 3. Results

This study included 100 participants, comprising 54 males and 46 females. The average age of the males and females was 16.63 years and 17.61 years, respectively, which was statistically significant (*p* = 0.002). The average IQ for males and females was 91.17 and 91.39, respectively, which was not statistically significant.

Among the 100 participants, 28 had ADHD, of whom 19 were female and 9 were male. Of the 19 women, 5 were hyperactivity-dominant, 4 were inattentive-dominant, and 1 exhibited a combination. Of the nine males, two were hyperactivity-dominant, four were inattentive-dominant, and three were combined (Table 1).

The psychiatric prevalence rate of the target group was examined using MINI-KID. Consequently, several diseases were found and arranged in descending order of their frequency (Table 2). These include (1) conduct disorder (CD), 49%; (2) alcohol dependence, 45%; (3) suicidal tendency, 34%; (4) ADHD, 28%; (5) alcohol abuse, 27%; (6) major depressive episode, past, 26%; (7) major depressive episode, present, 24%; (8) oppositional defiant disorder (ODD), 20%; (9) obsessive-compulsive disorder (OCD), 13%; (10) manic, present, hypomanic, past, 12%; (11) agoraphobia, 11%; (12) major depressive episode, recurrent, 10%; (13) post-traumatic stress disorder (PTSD), generalized anxiety disorder (GAD), 9%; (14) dysthymia, hypomania, present, 8%; (15) specific anxiety disorder, 7%; (16) separation anxiety disorder, 6%; (17) major depressive disorder, past, bipolar I past, panic, whole, social phobia, present, substance dependence, 5%; (18) major depressive disorder, present, manic, resent, mood disorder with psychotic features, whole, panic, past, social phobia, generalized, 4%; (19) bipolar I present, BN, psychotic disorder, whole, present, mood disorder with psychotic features, present, 3%; (20) bipolar II past, social phobia, nongeneralized, substance abuse, Tourette’s disorder, and motor, 2%; (21) bipolar II present, bipolar II past, unspecified bipolar, present, past, Pervasive Development Disorder (PDD), 1%; (22) major depressive disorder, recurrent, bipolar, 2 present, Tourette’s disorder, verbal Tourette’s disorder, temporary AN, adjustment disorder (AD), r/o, and medical or organic had a 0% prevalence.

Diseases that were more frequent in females are as follows (total %; F vs. M %): (1) alcohol dependence (45%; 56.52% vs. 35.12%); (2) major depressive episode, past (26; 30.43 vs. 22.22); (3) major depressive episode, present (24; 30.43 vs. 18.52); (4) ADHD, combined (13; 21.74 vs. 5.56); (5) OCD (13; 19.57 vs. 7.41); (6) mania, past (12; 15.22 vs. 9.26); (7) hypomania, past (12; 15.22 vs. 9.26); (8) agoraphobia, (11; 23.91 vs. 0.00); (9) major depressive episode, recurrent (10; 19.57 vs. 1.85); (10) GAD (9; 15.22 vs. 3.70); (11) PTSD (9; 17.39 vs. 1.85); (12) dysthymia (8; 13.04 vs. 3.70); (13) hypomania, present, (8; 8.70 vs. 7.41); (14) ADHD, inattentive, (8; 8.70 vs. 7.41); (15) specific phobia (7; 13.04 vs. 1.85); (16) ADHD, hyperactivity (7; 10.87 vs. 3.70); (17) separation anxiety disorder (6; 10.87 vs. 1.85); (18) panic, whole (5; 10.87 vs. 0.00); (19) social phobia, present (5; 10.87 vs. 0.00); (20) panic, present (4; 8.70 vs. 0.00); (21) social phobia, generalized (4; 4.35 vs. 3.70); (22) schizoaffective disorder, whole (4; 4.35 vs. 3.70); (23) social phobia, nongeneralized (unsporadic; 2; 4.35 vs. 0.00); (24) Tourette’s disorder, motor (2; 4.35 vs. 0.00); (25) substance abuse (2; 2.17 vs. 1.85); and (26) pervasive developmental disorders (PDD; 1.03; 2.33 vs. 0.00).

Diseases that were more frequent in males included (total %; F vs. M %) (1) CD (49; 41.30 vs. 55.56); (2) alcohol abuse (27; 6.52 vs. 44.44); (3) ODD (20; 17.39 vs. 22.22); (4) substance dependence (5; 4.35 vs. 5.56); (5) major depressive disorder, past, (5; 0.00 vs. 9.26); (6) bipolar type 1, past, (5; 0.00 vs. 9.26); (7) major depressive disorder, present (4; 0.00 vs. 7.41); 8) manic episode, present (4; 2.17 vs. 5.56); (9) bipolar type 1, present (3; 0.00 vs. 5.56); (10) schizoaffective disorder (i.e., mood disorder with psychotic features), present (3; 0.00 vs. 5.56); (11) schizophrenia (i.e., psychotic disorder), whole, (3; 2.17 vs. 3.70); (12) schizophrenia (i.e., psychotic disorder), present (3; 2.17 vs. 3.70); (13) bulimia nervosa, (3; 2.17 vs. 3.70); (14) bipolar type 2, past, (2; 0.00 vs. 3.70); (15) unspecified bipolar, present (1; 0.00 vs. 1.85); and (16) unspecified bipolar, past (1; 0.00 vs. 1.85).

Among them, 10 diseases were statistically significantly different between males and females. These included (1) recurrence of major depressive episodes, (2) panic disorder present, (3) panic disorder lifetime, (4) agoraphobia, (5) social phobia present, (6) specific phobia, (7) PTSD, (8) alcohol dependence, (9) alcohol abuse, and (10) ADHD combined type.

In terms of differences between males and females, only (9) alcohol abuse was higher in males, while in others, it was more frequent in females than in males.

Gender differences were investigated using MAYSI-2 (Table 3). Among each item, the items with a statistically significant gender difference were alcohol/drug use, feeling depressed/anxious, somatic complaints, suicidal ideation, and traumatic experiences. All items for which the gender difference was statistically significant were higher among females. Although not statistically significant, only angry/irritable items showed a high percentage in males.

## 4. Discussion

A high prevalence of psychopathology has been reported among prison inmates [2,19]. Adolescents in detention and correctional facilities are approximately 10 times more likely to suffer from psychosis than the general adolescent population [2].

Over the past decade, a gradual increase in the proportion of female offenders in the juvenile justice system has been observed. Shufelt et al. reported that more than 80% of the girls in their sample met the criteria for at least one disorder, in comparison to 67% of boys. This difference is mainly attributable to the increased rates of internalizing disorders (i.e., anxiety and mood disorders) among girls [20]. Thus, the study confirms previous findings of high rates of mental health disorders and suggests that the vast majority of youth involved in the juvenile justice system have several mental health disorders [20].

The main objectives of this study were to document the rate and prevalence of psychiatric disorders and the clinical characteristics of adolescents detained at a juvenile detention center in South Korea.

The psychiatric prevalence rate of the target group was examined using MINI-KID. Consequently, several diseases were found and arranged in descending order of their frequency, namely, CD (49%), alcohol dependence (45%), suicidal tendencies (34%), ADHD (28%), alcohol abuse (27%), major depressive episode, past (26%), major depressive episode, present (24%), ODD (20%), OCD (13%), and manic, present, hypomanic, past (12%). Many juvenile offenders in the current study had psychiatric disorders, including CD, alcohol dependence, suicidal tendencies, ADHD, and alcohol abuse as the most prevalent illnesses. Conduct disorder was the most common disorder, followed by alcohol dependence, suicidal tendencies, and ADHD. Furthermore, alcohol abuse, major depressive episodes, past, major depressive episodes, present, and ODD had a prevalence exceeding 20%. Choi et al. [21] investigated male adolescents in juvenile prisons in Korea and found that alcohol abuse (57.8%) was the most common disorder, followed by CD (55.5%), antisocial personality disorder (48%), bipolar disorder (47.4%), and ADHD (35.3%).

These findings are similar to the results of Collins et al., who found that the mean prevalence of any disorder was 69.9% (95% CI: 69.5–70.3), with CD occurring most frequently (46.4%; 95% CI: 45.6–47.3), followed by substance use disorder (45.1%; 95% CI: 44.6–45.5), ODD (19.8%; 95% CI: 9.2–20.3), and ADHD (13.5%; 95% CI: 13.2–13.9) [4]. In a meta-analysis by Fazel et al., high rates of psychotic illness (male adolescents, 3.3%), major depression (10.6%), ADHD (11.7%), and CD (52.8%) were described [2]. Despite the methodological differences between the two studies, the overall prevalence rates for ADHD (Fazel et al., 11.7%, compared with 28% in the current study), CD (52.8% vs. 49%), and major depression (10.6% vs. 24%) were similar [2].

As expected, CD was the most prevalent disorder, with a similar prevalence slightly higher than 49% [2]. CDs were nearly predominant in both male and female detainees [22]. The risk of CD is 5- to 10-fold higher than that in the general population [2]. The American Academy of Pediatrics report estimated the following prevalence ranges: 1% to 6% for psychosis, up to 50% for ADHD, and 20% to 60% for CD [23]. Some systematic reviews and meta-analyses found, on average, a fivefold increased prevalence of ADHD in youth prison populations (30.1%) and a tenfold increase in adult prison populations (26.2%) compared with published general population prevalence (i.e., 3–7% and 1–5%, respectively) [24].

Among these illnesses, 10 were statistically significantly different between males and females, namely, major depressive episodes (recurrent), panic disorder (present), panic disorder (lifetime), agoraphobia, social phobia (present), specific phobia, PTSD, alcohol dependence, alcohol abuse, and ADHD (combined type). In terms of differences between males and females, alcohol abuse was the only disease found at higher levels among males, whereas the remaining were more frequent in females. Consistent with previous studies that compared the prevalence of mental disorders among people in juvenile centers, the current study revealed a pattern indicating that women had a high prevalence of various mental disorders [7,25]. The current results are also consistent with research showing an increased need for mental health services among female juvenile offenders in contrast to their male counterparts [7,25]. Justices involving girls are associated with a higher risk for mental health disorders in contrast to cases involving boys [26]. Teplin et al. reported that nearly two-thirds of males and nearly three-quarters of females met the diagnostic criteria for at least one psychiatric disorder. Excluding CD (common among detained youth), nearly 60% of males and over 66% of females met the diagnostic criteria and had diagnosis-specific impairments for one or more psychiatric disorders [27]. Abram et al. reported that a significant proportion of females (56.5%) met the criteria for two or more of the following disorders, namely, major depressive, dysthymic, manic, psychotic, panic, separation anxiety, overanxious, generalized anxiety, OCD, attention-deficit hyperactivity, conduct, ODD, alcohol, marijuana, and other substances [28].

According to the National Comorbidity Study [29], men have an increased susceptibility to experiencing substance use and CDs than women, while women are much more likely to experience affective and anxiety disorders. Furthermore, the females were 2.5 to 4 times more likely than males to score in the moderate to severe range on the Major Depression and Dysthymic Disorder scales and were 1.4 times more likely than males to meet the criteria for an anxiety disorder. In a study by Robertson et al. [30], CD and substance abuse disorder were the only disorders with a higher prevalence in males than females. Furthermore, 50% of males and 39% of females met the criteria for CD. In this study, 55.6% and 41.3% of males and females, respectively, met the criteria for CD.

Among the MAYSI-2 items, the male and female differences were statistically significant, namely, alcohol or drug use, feeling depressed/anxious, somatic complaints, suicidal ideation, and traumatic experiences. The proportion of women was higher for all items where the gender difference was statistically significant. Although the difference was not statistically significant, only the angry/irritable items showed a high percentage of males. These results are somewhat similar to the findings of the MINI-KID used in this study that women exhibited increased levels of recurrent major depressive episodes, panic disorder, panic disorder lifetime, agoraphobia, social phobia, specific phobia, PTSD, alcohol dependence, and ADHD combined type, whereas alcohol abuse was the only disorder found in higher frequencies among the men.

Vincent et al. included data on MAYSI-2 scores for 70,423 youths from 283 juvenile justice probation, detention, or corrections programs. Across the sites, girls were generally 1.8 to 2.4 times more likely than boys to have clinical elevations on all applicable MAYSI-2 scales except the Alcohol/Drug Use scale. A sex effect was observed among the young individuals for the Alcohol/Drug Use scale [31].

Abram et al. examined suicidal ideation, suicide attempts, the lethality of suicide attempts, and the relationship between psychiatric disorders and recent attempts in newly detained juveniles. More than one-third of the juvenile detainees and nearly half of the females felt hopeless or contemplated death 6 months before detention. Approximately one in ten juvenile detainees had thought about committing suicide in the past 6 months, and one in ten had even attempted suicide. Recent suicide attempts were most prevalent in females and youth with major depression and GAD [32]. In Kroll et al.’s study, the prevalence of suicidal ideation in detainees who did not report self-harm (13.2%) was also higher than in the community sample (8.5%). This suggests that this group of incarcerated adolescents is at increased risk of suicidal behavior [33].

The current study found that the prevalence of various mental disorders was higher among female than male juvenile adolescents. Furthermore, this study confirmed that pathology has a higher severity in females. Therefore, females imprisoned in juvenile centers are more likely to have more serious crimes or pathologies due to the overall same-sex ratio as males. Girls who meet the criteria for CD have a higher risk of developing more severe psychopathology than their male counterparts [34,35]. Findings indicated that female adjudicated delinquents have significantly higher rates of psychopathology than males, suggesting an increased need for mental health services for girls in juvenile justice.

This study has several notable limitations. First, most of the detainees had high rates of psychiatric comorbidity. Consequently, there were insufficient detainees without psychiatric disorders to act as a control group. Therefore, further studies should include control groups to clarify the findings. This group can comprise detainees without any psychiatric disorder or adolescents recruited from the general population. Second, this study was conducted inside the detention center. Thus, the information may be limited, as the detainees were the only information sources for this study. Third, the participant pool in this study is somewhat limited in size. It is anticipated that an increase in the number of participants would yield richer results. Therefore, there is a need for further research involving a larger sample size. Fourth, in this paper, gender was represented solely as either male or female. This may be indicative of the conservative nature of gender identity expression in Korea, differing from Western norms. Future research should focus on a more detailed exploration of gender categories. Fifth, the detainees were obtained only from two detention centers. Therefore, large-scale studies including detainees from other areas and detention centers are required in the future. Finally, this paper, being conducted in South Korea, has inherent limitations in terms of global generalization and should be interpreted with caution regarding cultural diversity. Additionally, future research is necessary to compare the characteristics of different countries.

This study is significant because it is the first study in Korea to include both male and female adolescents in the context of juvenile school adolescents. Previous studies primarily focused on one gender (mainly male) as they are easily accessible. Furthermore, the prevalence was estimated using a semistructured questionnaire (i.e., MINI-KID) for all target groups. Although the MINI was used in previous studies by Choi et al. [21], it is significant as a follow-up study that compensates for the limitations of the age disparity present in some target groups of adolescents. Finally, the inclusion of both male and female participants may widen the generalizability of the current findings.

## 5. Conclusions

Juvenile detainees exhibited high rates of psychiatric disorders and comorbidities. Conduct disorders, alcohol dependence, and ADHD were the most common psychiatric disorders among juvenile detainees in South Korea. The assessment of and interventions in psychiatric disorders, especially CD, alcohol dependence, and ADHD, may help prevent further offenses. These findings highlight the importance of diagnosing and intervening in psychiatric disorders within juvenile detention systems.

## Figures and Tables

**Table 1:** Comparison of participant characteristics.

Variable	Level	Total (*n* = 100)	Female (*n* = 46)	Male (*n* = 54)	* *p*-Value	Test	t^2^/X^2^
Age (mean (sd))		17.08 (1.52)	17.61 (1.88)	16.63 (0.94)	0.00		3.21
FSIQ (mean (sd))		91.27 (8.35)	91.39 (6.38)	91.17 (9.78)	0.89		0.14

* *p* < 0.05; t^2^/X^2^ Chi-square; Abbreviations: SD, standard deviation; FSIQ, Full-Scale Intelligence Quotient.

**Table 2:** Prevalence of psychiatric disorders among detainees.

Variable	Level	Total (*n* = 100)	Sex = 0 (*n* = 46)	Sex = 1 (*n* = 54)	* *p*-Value	Test	t^2^/X^2^
Major depressive episode, present (%)					0.16		1.34
	0	76 (76.00)	32 (69.57)	44 (81.48)			
	1	24 (24.00)	14 (30.43)	10 (18.52)			
Major depressive episode, past (%)					0.35		0.50
	0	74 (74.00)	32 (69.57)	42 (77.78)			
	1	26 (26.00)	14 (30.43)	12 (22.22)			
Major depressive episode, recurrent (%)					0.01	exact	6.80
	0	90 (90.00)	37 (80.43)	53 (98.15)			
	1	10 (10.00)	9 (19.57)	1 (1.85)			
Major depressive disorder, present (%)					0.12	exact	1.88
	0	96 (96.00)	46 (100.00)	50 (92.59)			
	1	4 (4.00)	0 (0.00)	4 (7.41)			
Major depressive disorder, past (%)					0.06	exact	2.75
	0	95 (95.00)	46 (100.00)	49 (90.74)			
	1	5 (5.00)	0 (0.00)	5 (9.26)			
Major depressive disorder, recurrent (%)					-		
	0	100 (100)	46 (100.00)	54 (100.00)			
	1	0 (0.00)	0 (0.00)	0 (0.00)			
Suicidal tendency (%)					0.12		4.19
	low	26 (43.33)	11 (32.35)	15 (57.69)			
	medium	10 (16.67)	6 (17.65)	4 (15.38)			
	high	24 (40.00)	17 (50.00)	7 (26.92)			
Suicidal tendency (%)					0.01	exact	11.01
	none	40 (40.00)	12 (26.09)	28 (51.85)			
	low	26 (26.00)	11 (23.91)	15 (27.78)			
	medium	10 (10.00)	6 (13.04)	4 (7.41)			
	high	24 (24.00)	17 (36.96)	7 (12.96)			
Dysthymia (%)					0.14	exact	1.81
	0	92 (92.00)	40 (86.96)	52 (96.30)			
	1	8 (8.00)	6 (13.04)	2 (3.70)			
Manic episode, present (%)					0.62	exact	0.12
	0	96 (96.00)	45 (97.83)	51 (94.44)			
	1	4 (4.00)	1 (2.17)	3 (5.56)			
Manic episode, past (%)					0.36		0.37
	0	88 (88.00)	39 (84.78)	49 (90.74)			
	1	12 (12.00)	7 (15.22)	5 (9.26)			
Hypomanic episode, present (%)					1.00	exact	0.00
	0	92 (92.00)	42 (91.30)	50 (92.59)			
	1	8 (8.00)	4 (8.70)	4 (7.41)			
Hypomanic episode, past (%)					0.36		0.37
	0	88 (88.00)	39 (84.78)	49 (90.74)			
	1	12 (12.00)	7 (15.22)	5 (9.26)			
Bipolar I disorder, present (%)					0.25	exact	1.07
	0	97 (97.00)	46 (100.00)	51 (94.44)			
	1	3 (3.00)	0 (0.00)	3 (5.56)			
Bipolar I disorder, past (%)					0.06	exact	2.75
	0	95 (95.00)	46 (100.00)	49 (90.74)			
	1	5 (5.00)	0 (0.00)	5 (9.26)			
Bipolar II disorder, present (%)					-		
	0	100 (100.00)	46 (100.00)	54 (100.00)			
	1	0 (0.00)	0 (0.00)	0 (0.00)			
Bipolar II disorder, past (%)					0.50	exact	0.36
	0	98 (98.00)	46 (100.00)	52 (96.30)			
	1	2 (2.00)	0 (0.00)	2 (3.70)			
Bipolar disorder unspecified, present (%)					1.00	exact	0.00
	0	99 (99.00)	46 (100.00)	53 (98.15)			
	1	1 (1.00)	0 (0.00)	1 (1.85)			
Bipolar disorder unspecified, past (%)					1.00	exact	0.00
	0	99 (99.00)	46 (100.00)	53 (98.15)			
	1	1 (1.00)	0 (0.00)	1 (1.85)			
Panic disorder, present (%)					0.04	exact	2.89
	0	96 (96.00)	42 (91.30)	54 (100.00)			
	1	4 (4.00)	4 (8.70)	0 (0.00)			
Panic disorder, whole (%)					0.02	exact	4.10
	0	95 (95.00)	41 (89.13)	54 (100.00)			
	1	5 (5.00)	5 (10.87)	0 (0.00)			
Agoraphobia (%)					<0.001		12.17
	0	89 (89.00)	35 (76.09)	54 (100.00)			
	1	11 (11.00)	11 (23.91)	0 (0.00)			
Separation anxiety disorder (%)					0.09	exact	2.16
	0	94 (94.00)	41 (89.13)	53 (98.15)			
	1	6 (6.00)	5 (10.87)	1 (1.85)			
Social phobia, present (%)					0.02	exact	4.10
	0	95 (95.00)	41 (89.13)	54 (100.00)			
	1	5 (5.00)	5 (10.87)	0 (0.00)			
Social phobia, sporadic (%)					1.00	exact	0.00
	0	96 (96.00)	44 (95.65)	52 (96.30)			
	1	4 (4.00)	2 (4.35)	2 (3.70)			
Social phobia, unsporadic (%)					0.21	exact	0.69
	0	98 (98.00)	44 (95.65)	54 (100.00)			
	1	2 (2.00)	2 (4.35)	0 (0.00)			
Specific phobia (%)					0.05	exact	3.21
	0	93 (93.00)	40 (86.96)	53 (98.15)			
	1	7 (7.00)	6 (13.04)	1 (1.85)			
Obsessive-compulsive disorder (%)					0.07		2.26
	0	87 (87.00)	37 (80.43)	50 (92.59)			
	1	13 (13.00)	9 (19.57)	4 (7.41)			
Post-traumatic stress disorder (%)					0.01	exact	5.55
	0	91 (91.00)	38 (82.61)	53 (98.15)			
	1	9 (9.00)	8 (17.39)	1 (1.85)			
Alcohol dependence (%)					0.03		3.75
	0	55 (55.00)	20 (43.48)	35 (64.81)			
	1	45 (45.00)	26 (56.52)	19 (35.19)			
Alcohol abuse (%)					<0.001		16.25
	0	73 (73.00)	43 (93.48)	30 (55.56)			
	1	27 (27.00)	3 (6.52)	24 (44.44)			
Material dependence (%)					1.00	exact	0.00
	0	95 (95.00)	44 (95.65)	51 (94.44)			
	1	5 (5.00)	2 (4.35)	3 (5.56)			
Material abuse (%)					1.00	exact	0.00
	0	98 (98.00)	45 (97.83)	53 (98.15)			
	1	2 (2.00)	1 (2.17)	1 (1.85)			
Tourette’s disorder, motor (%)					0.21	exact	0.69
	0	98 (98.00)	44 (95.65)	54 (100.00)			
	1	2 (2.00)	2 (4.35)	0 (0.00)			
Tourette’s disorder, vocal (%)					-		
	0	100 (100.00)	46 (100.00)	54 (100.00)			
	1	0 (0.00)	0 (0.00)	0 (0.00)			
Tourette’s disorder, temporary (%)					-		
	0	100 (100.00)	46 (100.00)	54 (100.00)			
	1	0 (0.00)	0 (0.00)	0 (0.00)			
ADHD, combined (%)					0.02		4.41
	0	87 (87.00)	36 (78.26)	51 (94.44)			
	1	13 (13.00)	10 (21.74)	3 (5.56)			
ADHD, AD dominant (%)					1.00	exact	0.00
	0	92 (92.00)	42 (91.30)	50 (92.59)			
	1	8 (8.00)	4 (8.70)	4 (7.41)			
ADHD, HA dominant (%)					0.24	exact	1.01
	0	93 (93.00)	41 (89.13)	52 (96.30)			
	1	7 (7.00)	5 (10.87)	2 (3.70)			
Conduct disorder (%)					0.16		1.49
	0	51 (51.00)	27 (58.70)	24 (44.44)			
	1	49 (49.00)	19 (41.30)	30 (55.56)			
ODD (%)					0.55		0.12
	0	80 (80.00)	38 (82.61)	42 (77.78)			
	1	20 (20.00)	8 (17.39)	12 (22.22)			
Schizophrenia, whole (%)					1.00	exact	0.00
	0	97 (97.00)	45 (97.83)	52 (96.30)			
	1	3 (3.00)	1 (2.17)	2 (3.70)			
Schizophrenia, present (%)					1.00	exact	0.00
	0	97 (97.00)	45 (97.83)	52 (96.30)			
	1	3 (3.00)	1 (2.17)	2 (3.70)			
Schizoaffective disorder, whole (%)					1.00	exact	0.00
	0	96 (96.00)	44 (95.65)	52 (96.30)			
	1	4 (4.00)	2 (4.35)	2 (3.70)			
Schizoaffective disorder, present (%)					0.25	exact	1.07
	0	97 (97.00)	46 (100.00)	51 (94.44)			
	1	3 (3.00)	0 (0.00)	3 (5.56)			
Anorexia nervosa (%)					-		
	0	100 (100.00)	46 (100.00)	54 (100.00)			
	1	0 (0.00)	0 (0.00)	0 (0.00)			
Bulimia nervosa (%)					-		
	0	97 (97.00)	45 (97.83)	52 (96.30)			
	1	3 (3.00)	1 (2.17)	2 (3.70)			
Generalized anxiety disorder (%)					0.08	exact	2.74
	0	91 (91.00)	39 (84.78)	52 (96.30)			
	1	9 (9.00)	7 (15.22)	2 (3.70)			
Adjustment disorder (%)					-		
	0	100 (100.00)	46 (100.00)	54 (100.00)			
	1	0 (0.00)	0 (0.00)	0 (0.00)			
Other medical/material/drug cause (%)					-		
	0	100 (100.00)	46 (100.00)	54 (100.00)			
	1	0 (0.00)	0 (0.00)	0 (0.00)			
Pervasive developmental disorder (%)					0.44	exact	0.01
	0	96 (98.97)	42 (97.67)	54 (100.00)			
	1	1 (1.03)	1 (2.33)	0 (0.00)			

* *p* < 0.05. t^2^/X^2^ Chi-square, independent *t*-test for continuous variables. Chi-square test or Fisher’s exact test for categorical variables. Abbreviations: OCD, obsessive-compulsive disorder; ADHD, attention-deficit/hyperactivity disorder; AD, adjustment disorder; ODD, oppositional defiant disorder.

**Table 3:** Gender differences in MAYSI among detainees.

Variable	Total (*n* = 100)	F (*n* = 46)	M (*n* = 54)	* *p*-Value
BAD (mean (sd))	2.21 (2.14)	3.5 (2.00)	1.11 (1.57)	<0.001
Alcohol/drug use (mean (sd))	3 (2.78)	4.7 (2.53)	1.56 (2.10)	<0.001
BAI (mean (sd))	3.14 (2.63)	3.04 (2.43)	3.22 (2.81)	0.734
Angry/irritable (mean (sd))	3.54 (2.95)	3.22 (2.67)	3.81 (3.16)	0.312
Depressed/anxious(mean (sd))	3.21 (2.72)	4.67 (2.29)	1.96 (2.43)	<0.001
Somatic complaints (mean (sd))	1.88 (2.02)	2.78 (1.87)	1.11 (1.82)	<0.001
Suicidal ideation (mean (sd))	1.49 (1.89)	2.48 (1.95)	0.65 (1.38)	<0.001
Thought disturbance (mean (sd))	2.21 (2.29)	4.41 (1.20)	0.33 (0.89)	<0.001

* *p* < 0.05. Abbreviations: MAYSI, Massachusetts Youth Screening Instrument; BAD, bipolar affective disorder; BAI, Beck Anxiety Inventory.

## Data Availability

Data are contained within the article.
